# Synergistic Effect of Ferulic Acid and Z-Ligustilide, Major Components of* A. sinensis*, on Regulating Cold-Sensing Protein TRPM8 and TPRA1* In Vitro*

**DOI:** 10.1155/2016/3160247

**Published:** 2016-06-20

**Authors:** Yuwei Pan, Guoping Zhao, Zejian Cai, Fengguo Chen, Dandan Xu, Si Huang, Hai Lan, Yi Tong

**Affiliations:** ^1^Department of Traditional Chinese Medicine, College of Medicine, Jinan University, Guangzhou 510630, China; ^2^Shipai District Community Health Service Center, Guangzhou 510630, China; ^3^Guangdong Food and Drug Vocational College, Guangzhou 510520, China

## Abstract

*Angelica sinensis* has been used to attenuate cold-induced cutaneous vasospasm syndrome, such as Raynaud's disease and frostbite, in China for many years. Ferulic acid (PubChem CID: 445858) and Z-ligustilide (PubChem CID: 529865), two major components extracted from* Angelica sinensis*, had been reported to inhibit vasoconstriction induced by vasoconstrictors. In this study, the pharmacological interaction in regulating cold-induced vascular smooth muscle cell contraction via cold-sensing protein TRPM8 and TRPA1 was analyzed between ferulic acid and Z-ligustilide. Pharmacological interaction on inhibiting [Ca^2+^]_i_ influx evoked by TRPM8 agonist WS-12 or TRPA1 agonist ASP 7663 as well as cold-induced upregulation of TRPM8 was determined using isobolographic analysis. The isobolograms demonstrated that the combinations investigated in this study produced a synergistic interaction. Combination effect of two components in inhibiting RhoA activation and phosphorylation of MLC_20_ induced by WS-12 or ASP 7663 was also being quantified. These findings suggest that the therapeutic effect of* Angelica sinensis* on cold-induced vasospasm may be partially attributed to combinational effect, via TRPM8 and TPRA1 way, between ferulic acid and Z-ligustilide.

## 1. Introduction

The roots of* Angelica sinensis* (RAS) are a well-known traditional Chinese medicine which had been used for thousands of years in China. RAS is widely accepted for its anticancer, memory, neuroprotective, and immunoregulatory effects [1]. It has also been used to cure some cold-induced cutaneous vasospasm diseases such as Raynaud's phenomenon and frostbite [2]. Ferulic acid and Z-ligustilide, which proved to be effective in cardiovascular disease, are two major components extracted from* Angelica sinensis* [3, 4]. However, mechanism of RAS in regulating cold-induced vasospasm is still unknown.

Ion channels, especially Ca^2+^-permeable channels, play an important role in regulating vascular tone [5]. Members of transient receptor potential (TRP) family that are considered as novel nonvoltage Ca^2+^ permeable cation channels attract people's attention in vascular research. Several members of TRP family exhibit temperature-sensitive characters [6]. TRP channels, expressed in sensory neurons of dorsal root ganglia (DRG) and trigeminal ganglia (TG), are the primary detectors for sensing environmental change and stimuli [7]. Among them, TRPM8 and TRPA1 are sensors for noxious cold and innocuous cool temperature, respectively [6, 8, 9]. TRPM8 can be activated by some cooling compounds, such as menthol or icilin, while TRPA1 can be activated by mustard oil or AITC. Recently, several studies proved that TRPM8 and TRPA1 not only existed in sensory neurons but also could be detected in rat aorta and pulmonary artery [10]. Both of them are involved in the regulation of vascular tone, especially response to cold environment [5, 11, 12].

Local cold exposure leads to an initial vasoconstriction which protects against heat loss, followed by a vasodilation which prevent local area from cold-induced injuries such as frostbite [13]. The complex regulator mechanisms involve a combination of neurotransmitter synthesis and release, Ca^2+^ homeostasis, adrenergic receptor function, or VSMC contractile ability [14]. Sympathetic nerve, sensory nerve, and nonneuronal factors, such as NOS system, contributed to the cutaneous vasoconstrictor response to local cooling [14, 15]. Contraction of vascular smooth muscle cell (VSMC), influenced by systems mentioned above, controlled vascular tone directly. Constriction of VSMC can be achieved by an increase of intracellular Ca^2+^ influx, which is recognized as “calcium-dependent,” as well as an increase of Ca^2+^ sensitivity, which is recognized as “calcium sensitization” [16]. TRPM8 and TRPA1 could regulate vascular tone through sensory nerve, sympathetic nerve, and NOS system [12, 17].

In order to discuss TRPM8 and TRPA1 direct effect on vascular smooth muscle cell contraction, TRPM8 specific agonist WS-12 and TRPA1 specific agonist ASP 7663 were used. Both “calcium-dependent” pathway and “calcium-sensitization” pathway were explored. Since cold temperature can activate TRPM8 and TRPA1 ion channel, its regulation on TRPM8 and TRPA1 expression was discussed as well. Since prolong exposure to cold environment leads to some local area cold-induced injuries which could be attenuated by application of* Angelica sinensis*, synergistic effect of ferulic acid and Z-ligustilide on regulating TRPM8/TRPA1 function and expression would be discussed.

## 2. Materials and Methods

### 2.1. Reagents

Ferulic acid and Z-ligustilide were obtained from National Institutes for food And Drug Control (Beijing, China). WS-12, ASP 7663, ryanodine, 2-APB, and xestospongin C were acquired from Tocris Bioscience (Bristol, United Kingdom). Fluo-4, AM, cell permeable, and pluronic F-127 were purchased from Thermo Fisher Scientific (Waltham, MA, USA). Rhotekin-RBD bead pulldown kit was from Cytoskeleton (St. Denver, CO, USA). Primary antibodies against MLC and phosphor-MLC were from Cell Signaling Technology (Beverly, MA, USA).

### 2.2. Cell Culture

The human aortic smooth muscle cell (ScienCell, Carlsbad, CA, USA) was cultured in smooth muscle cell medium (ScienCell, Carlsbad, CA, USA) containing 10% FBS and antibiotics (100 U/mL penicillin and 100 *μ*g/mL streptomycin) at 37°C in a humidified incubator with 5% CO_2_.

### 2.3. Calcium Imaging

Human aortic smooth muscle cells on coverslip were loaded with 5 *μ*M fluo-4 and 0.01% pluronic acid in HBSS for 60 min at 37°. Coverslip was placed on fluorescence microscope (Olympus IX71, Xuhui District, Shanghai). Fluorescence signal was measured at 561 nm. Images were captured every 4 s for 15 min with a ×5 objective lens and analyzed using ImagJ software. For each coverslip at least 50 cells in each image were included as a region of interest (ROI). The average intensity of ROIs was calculated at each time-point. The highest fluorescence (*F*_max_) was recognized as the maximal response to agonist WS-12 or ASP 7663 (50 *μ*M; set at 100%) whereas the baseline fluorescence (*F*_0_) was the weakest and stable fluorescence before agonist added. Change of fluorescence *F*_max_/*F*_0_ was recorded.

### 2.4. RNA Extraction and RT-qPCR Analysis

Cells were seeded at a density of 2 × 10^5^ cells/dish and incubated with ferulic acid, Z-ligustilide, or their combination in the presence of cold stimulation. RNA was extracted from HASMC using Trizol reagent (Invitrogen Life Technologies, Darmstadt, Germany) under manufacturer's instructions. The RNA quantity was estimated by a nanodrop spectrophotometer (Thermo Scientific, Rockford, IL). cDNA was made from 500 ng of total RNA using PrimeScript*™* RT Master Mix (TAKARA, Kyoto, Japan). The resulting cDNA was used to perform real-time fluorescence quantitative PCR reactions in triplicate using SsoFast*™* EvaGreen® Supermixes (Bio-Rad, California, USA) and CFX Connect*™* Real-Time PCR Detection System (Bio-Rad, California, USA) operated by CFX Manager Software. RTFQ PCR was initiated with 1 × 98°C for 2 minutes, followed by 39 cycles of 98°C for 2 seconds and 58°C for 5 seconds, and finalized with melt curve for 75°C to 95°C for 10 seconds. Forward and reverse primers were designed and ordered from Sangon Biotech (Songjiang, Shanghai, China). *β*-actin was used as reference gene. Primer sequences were as follows: TRPM8 forward CAG CAC TGG CAC CTG AAA AC; TRPM8 reverse GGA CTG CGC GAT GTA GAT GA; TRPA1 forward GCT ACT CTC TAA AGG TGC CCA AG; TPRA1 reverse CGT TGT CTT CAT CCA TTA CCA G; *β*-actin forward GGG AAA TCG TGC GTG ACA TTA AGG; *β*-actin reverse CAG GAA GGA AGG CTG GAA GAG TG. The expression levels of the target genes were normalized to *β*-actin.

### 2.5. Western Blotting

Lysis of HASMC was made in RIPA including protease inhibitor purchased from Beyotime Biotechnology (Songjiang, Shanghai, China). Lysate concentration was determined using BCA assay and a standard curve. Equal amounts were loaded on 8%–12% Bis-Tris gels, subjected to electrophoresis, and transferred to polyvinylidene difluoride (PVDF) membranes. Membranes were probed with appropriate primary antibodies and then incubated with the horse-radish peroxidase-conjugated secondary antibodies. Immunoreactive bands were visualized using the chemiluminescence detection kit (CST, Beverly, MA, USA).

### 2.6. Detection of Total and GTP-Bound RhoA

HSMAC were seeded in 60 mm tissue culture dish and grow to 30% confluence. Then they were made quiescent by serum starvation (0% FBS) for 24 hours. Active and GTP-bound RhoA were measure from cell lysates using Rhotekin-RBD bead pulldown assay according to the manufacturer's instruction. Total RhoA was determined by Western blot with an anti-RhoA and was used to normalize GTP-RhoA expression. Activated RhoA in stimulated samples was compared to the level of GTP-RhoA in unstimulated samples.

### 2.7. Analysis of Combined Drug Effects

We used the Calcusyn software (Biosoft, Version 2.1) to determine drug synergy by the isobolograms and combination index originated from the median effect principle of Chou and Talalay [18]. The isobolograms method, which is formed by selecting a desired fractional affect (Fa), represents the pharmacologic interaction. A straight line was drawn to connect the Fa points plotted with fixed ration between ferulic acid and Z-ligustilide on *x*- and *y*-axes to generate isobolograms. Combination data points falling on the line represented an additive drug-drug interaction while points falling below or above the line represented synergism or antagonism. The combination index (CI) was calculated by the following formula [19]: CI = (*D*)_A_/(*D*_*x*_)_A_ + (*D*)_B_/(*D*_*x*_)_B_. (*D*)_A_ and (*D*)_B_ were the concentrations of drug A and drug B, used in combination to achieve *x*% drug effect. (*D*_*x*_)_A_ and (*D*_*x*_)_B_ were the concentrations of individual components to achieve the same effect. CI values were generated over a range of Fa levels from 0.05 to 0.90 (5%–90% inhibition). CI of 1 indicated additive effect between the two components whereas CI < 1 or CI > 1 indicated synergism or antagonism separately.

### 2.8. Statistical Analysis

Each experiment was performed using 3 individual coverslips of cells and data are presented as mean ± SEM. Data statistical analysis was performed using two-tailed Student's *t*-test or one-way analysis of variance followed by the SNK* post hoc* test. *P* values of <0.05 were considered statistically significant.

## 3. Results and Discussion

### 3.1. Responses to TRPM8 and TRPA1 Agonist-Evoked Ca^2+^ Signal

#### 3.1.1. Contribution of Ca^2+^ Store to TRPM8 and TRPA1 Agonist-Evoked Ca^2+^ Signal

**Figure 1:**
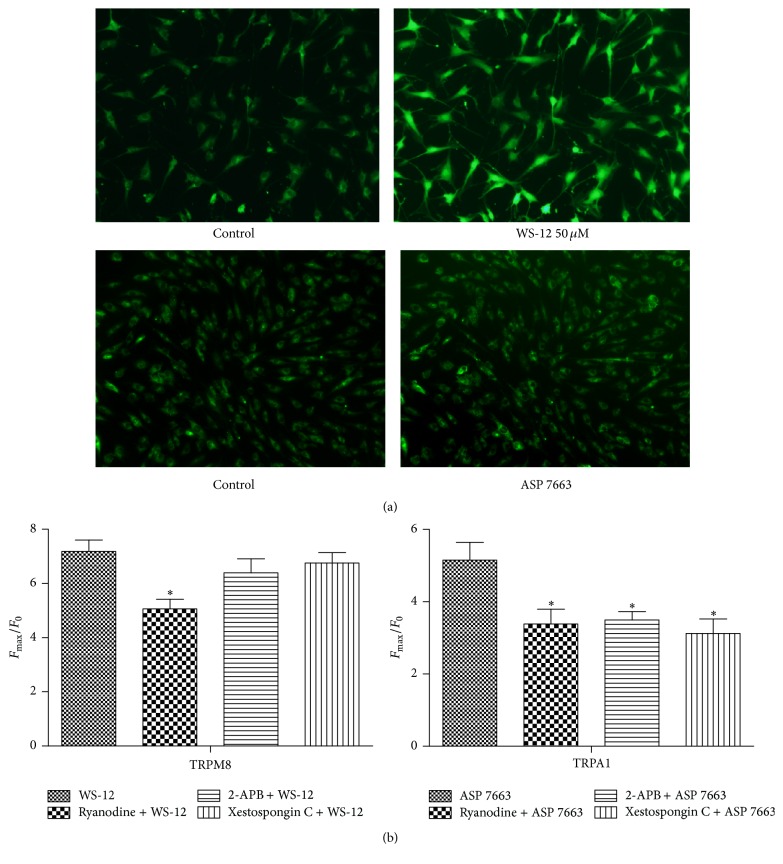
Contribution of Ca^2+^ store to TRPM8 and TRPA1 agonist-evoked Ca^2+^ signal. (a) Images of field of HASMC before (left) and during (right) 50 *μ*M WS-12 (upper) and 50 *μ*M ASP7663 (down) application. (b) WS-12 50 *μ*M or ASP 50 *μ*M-evoked calcium signal was measured in the absence or presence of ryanodine 30 *μ*M or 2-APB 100 *μ*M or xestospongin C 5 *μ*M.  ^*∗*^Significant difference between control groups (*P* < 0.05).

We first examined the effects of TRPM8 specific agonist WS-12 50 *μ*M (*F*_max_/*F*_0_ = 7.186 ± 0.8334) and TRPA1 specific agonist ASP 7663 50 *μ*M (*F*_max_/*F*_0_ = 5.153 ± 0.9831). As illustrated in Figure 1(a), both of them could induce significant change of fluorescence.

TRPM8 and TRPA1 were Ca^2+^ permeable cation channels which could activate calcium induced calcium release (CICR) through either ryanodine receptor (RYR) or inositol trisphosphate receptor (IP_3_R). To assess if Ca^2+^ release via RYR and IP_3_R contributes to TRPM8 and TRPA1 agonist-evoked Ca^2+^ signal, experiments were carried out with the presence of RYR inhibitor or IP_3_R inhibitor. As shown in Figure 1(b), application of a high concentration of RYR inhibitor ryanodine (30 *μ*M) could significantly reduce both ASP 7663 (*F*_max_/*F*_0_ = 3.384 ± 0.8142) and WS-12 (*F*_max_/*F*_0_ = 5.066 ± 0.6962) evoked [Ca^2+^]_i_, while application of IP_3_R inhibitor 2-APB (100 *μ*M) or xestospongin C (5 *μ*M) could only reduce ASP 7663 (*F*_max_/*F*_0_ = 3.499 ± 0.4609 for 2-APB and *F*_max_/*F*_0_ = 3.122 ± 0.6891 for xestospongin C) but not WS-12 (*F*_max_/*F*_0_ = 6.395 ± 1.030 for 2-APB and *F*_max_/*F*_0_ = 6.76 ± 0.6533 for xestospongin C) evoked [Ca^2+^]_i_. These data indicated that Ca^2+^ release via RYR contributes to TRPM8 agonist-evoked Ca^2+^ signal, while Ca^2+^ release via both RYR and IP_3_R contributes to TRPA1 agonist-evoked Ca^2+^ signal.

#### 3.1.2. Inhibition of WS-12 and ASP 7663 by Ferulic Acid and Z-Ligustilide Individually

**Figure 2:**
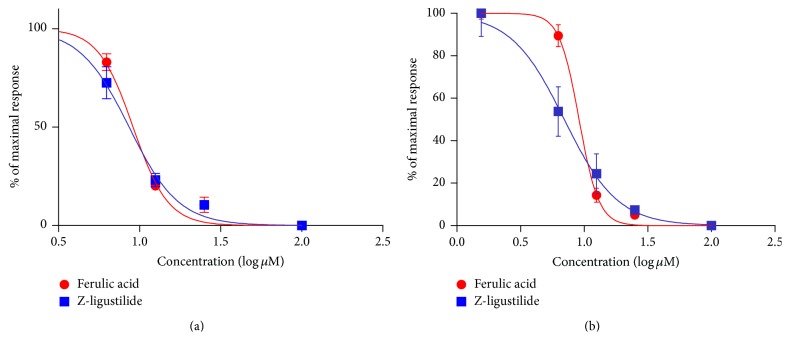
Effects of ferulic acid and Z-ligustilide on [Ca^2+^]_i_ entry responses to TRPM8 or TRPA1 agonist with absence of CICR. Inhibition of HASMC response to WS-12 50 *μ*M (a) or ASP 7663 50 *μ*M (b) by ferulic acid (red symbols and line) or Z-ligustilide (blue symbols and line) with absence of CICR. Sigmoidal log concentration-response curve was drawn. Results were presented as a percentage of the peak [Ca^2+^]_i_ entry level measured in the presence of 50 *μ*M WS-12 or ASP 7663 50 *μ*M. Each value was derived from 3 individual experiments with a total of 30–80 cells. HASMC were quiescent in serum-free medium for 24 hours before experiments.

To exclude calcium release through CICR, 10 minutes prior to the challenge with WS-12, cells were first perfused with ryanodine 30 *μ*M. Meanwhile, 10 minutes prior to the challenge with ASP, cells were first perfused with combination of ryanodine 30 *μ*M and 2-APB 100 *μ*M. Next, ferulic acid and Z-ligustilide in the concentrations (*μ*M) 1.55, 6.25, 12.5, 25, and 100 followed by WS-12 50 *μ*M or ASP 7663 50 *μ*M were applied. The concentration-response curve can be seen in Figure 2. The fact that increasing concentrations of compounds related to decreasing [Ca^2+^]_i_ release showed both drugs inhibiting the TRPM8 activated by WS-12 or TRPA1 activated by ASP 7663 in the absence of CICR.

#### 3.1.3. Inhibition of WS-12 and ASP 7663 by the Drug Combination

**Figure 3:**
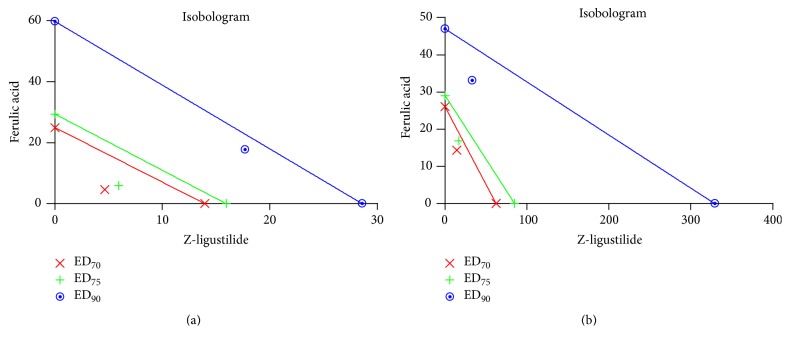
Isobolograms of combination of ferulic acid with Z-ligustilide in inhibiting [Ca^2+^]_i_ response to agonists. The individual doses of ferulic acid and Z-ligustilide to achieve 90% (straight line) inhibition (Fa = 0.90), 75% (hyphenated line) inhibition (Fa = 0.75), and 70% (crosses) inhibition (Fa = 0.70) were plotted on the *x*-axes and *y*-axes. Combination index (CI) values calculated by Calcusyn software are represented by points above (indicating antagonism) or below the lines (indicating synergism).

The individual IC_50_ values for ferulic acid (8.318 ± 1.03 *μ*M) and Z-ligustilide (7.839 ± 1.09 *μ*M) were used to define four concentrations of the drugs in the combination at a fixed ratio for inhibition of WS-12, while ferulic acid (9.124 ± 1.04 *μ*M) and Z-ligustilide (6.83 ± 1.12 *μ*M) were used in the same way for inhibition of ASP 7663 (Table 1). Combination index was used to confirm the synergistic effect of ferulic acid with Z-ligustilide. The CI values were calculated by Calcusyn software to analyze the degree of drug interaction. Isobolograms and Fa values were provided to descript the inhibition rate at Figure 3 and Table 2. The CI values of <1 and >1 represented synergistic and antagonistic effect separately. Since all the CI values were <1, our results showed ferulic acid and Z-ligustilide having synergistic effect to inhibit [Ca^2+^]_i_ release from HASMC exposing to TRPM8 agonist WS-12 or TRPA1 agonist ASP 7663.

### 3.2. Responses to WS-12 or ASP 7663-Induced RhoA Activation and MLC_20_ Phosphorylation

**Figure 4:**
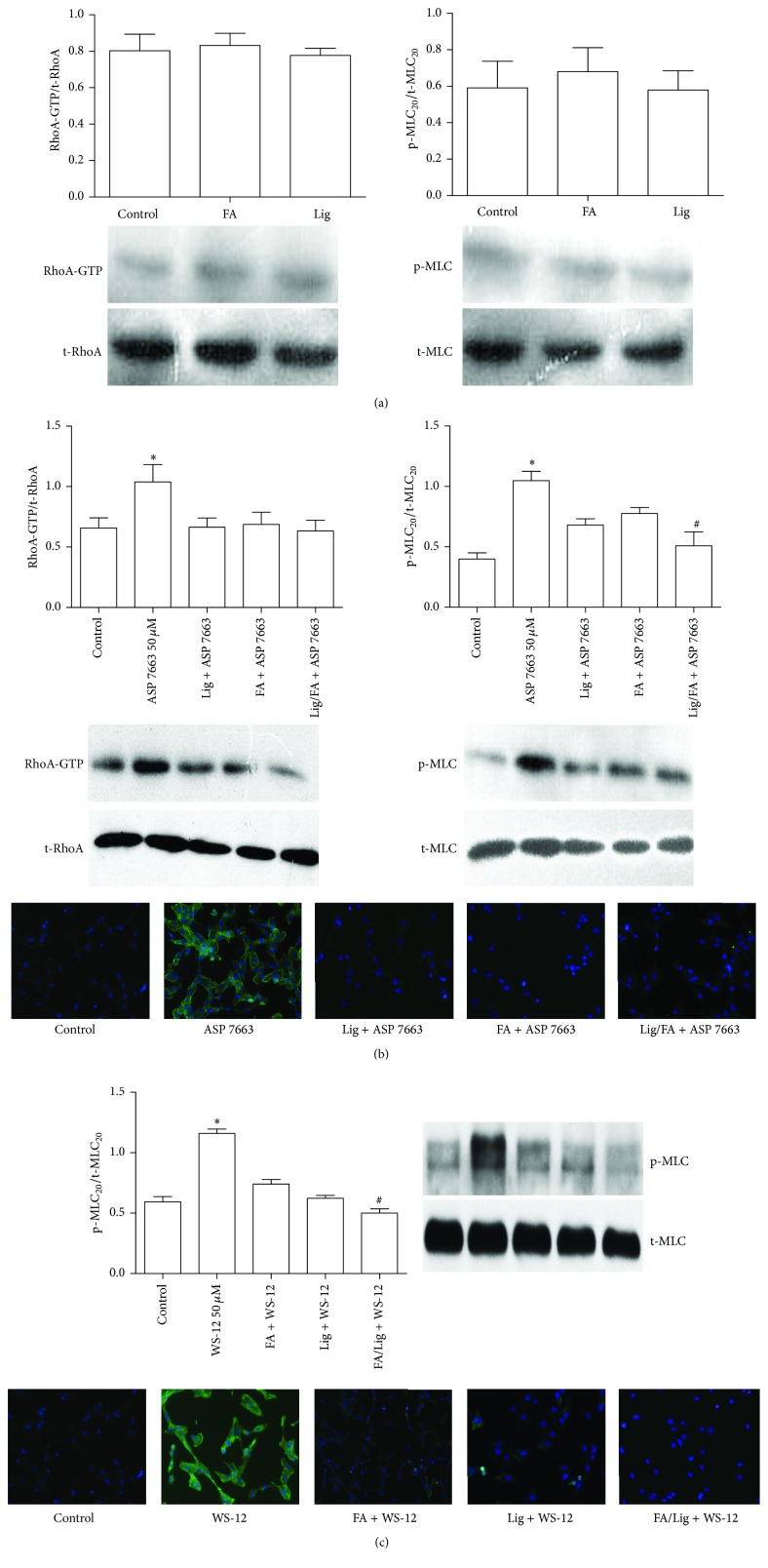
Inhibition effects of ferulic acid, Z-ligustilide, or their combination on GTP-RhoA level and phosphorylation of MLC_20_ with absence or presence of WS-12 or ASP 7663. HASMC were stimulated by ferulic acid or Z-ligustilide alone (a), 50 *μ*M WS-12 (b), or 50 *μ*M ASP 7663 (c) for 10 minutes in the absence or presence of ferulic acid, Z-ligustilide, or their combination. Data are expressed in % of GTP-RhoA expression normalized to total RhoA expression or phosphorylation of MLC_20_ normalized to total MLC_20_ expression. Immunofluorescence images of phosphorylation of MLC_20_ were also captured. Bars are mean values from 3 individual samples.  ^*∗*^Significant difference compared to control (*P* < 0.05).  ^#^Significant difference compared to ferulic acid group or Z-ligustilide group (*P* < 0.05).

To determine whether ferulic acid and Z-ligustilide may influence calcium sensitivity and eventually smooth muscle cell contraction, via TRPM8 channel or TRPA1 channel, HASMC were tested in the presence or absence of drugs for the occurrence of WS-12 or ASP 7663 induced RhoA activation and phosphorylation of MLC_20_ by Western blotting. Concentration of ferulic acid, Z-ligustilide, and their combination was determined by IC_50_ obtained from experiment above. Ferulic acid or Z-ligustilide could activate neither RhoA nor phosphorylation of MCL_20_ in HASCM without any stimulation (Figure 4(a)). ASP 7663 treatment increased the GTP-RhoA level and phosphorylation of MLC_20_ signal, while ferulic acid (18 *μ*M), Z-ligustilide (13 *μ*M), and their combination (31 *μ*M) could all decrease it (Figure 4(b)). WS-12 treatment could not increase GTP-RhoA level (Data not shown), while it could still increase phosphorylation of MLC_20_ signal. Ferulic acid (16 *μ*M), Z-ligustilide (15 *μ*M), and their combination (31 *μ*M) can all decrease it (Figure 4(c)).

### 3.3. Response to Cold-Induced Upregulation of TRPM8 and TRPA1

**Figure 5:**
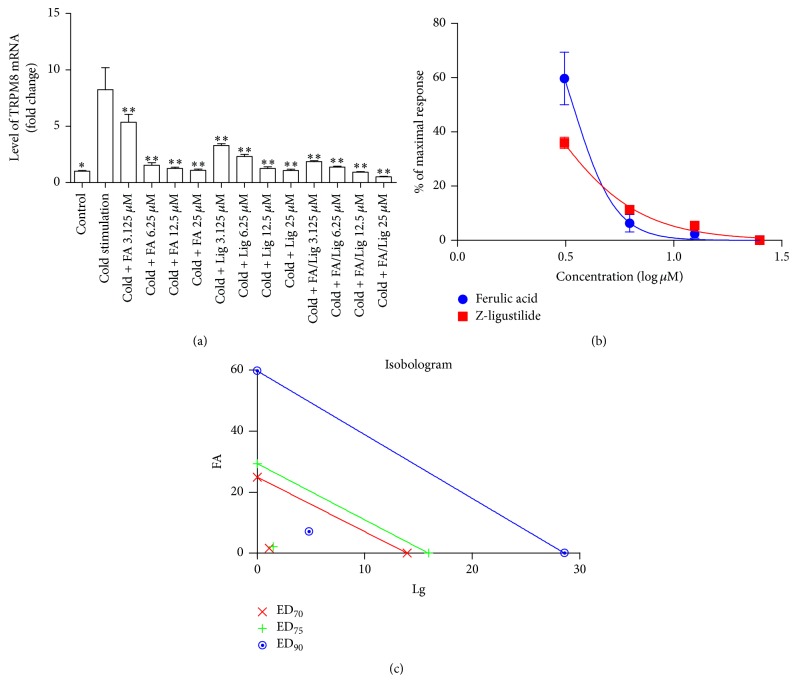
Effect of ferulic acid, Z-ligustilide, or their combination on cold-induced upregulation of TRPM8. (a) and (b) show effects of different concentration of ferulic acid and Z-ligustilide on inhibiting* trpm8* gene expression. (c) shows isobolograms of combination of ferulic acid with Z-ligustilide. Combination index (CI) values calculated by Calcusyn software were all below points, which indicate synergism.  ^*∗*^Significant difference between control group versus cold-stimulation group.  ^*∗∗*^Significant difference between groups versus cold-stimulation group.

Figure 5(a) showed higher gene expression of TRPM8 from HASMC incubating at 10°C (5% CO_2_) for 4 hours, when compared to control group. TRPA1 gene expression was not influenced by 10° (Data not shown). Different concentration of ferulic acid and Z-ligustilide could inhibit upregulation of TRPM8 mRNA expression (Figure 5(a)). The individual IC_50_ values (Figure 5(b)) of ferulic acid (3.416 ± 1.04 *μ*M) and Z-ligustilide (2.325 ± 1.05 *μ*M) were used to define four concentrations (Table 3) of the drugs in the combination at a fixed ratio for response to cold-induced upregulation of TRPM8. Isobolograms (Figure 5(c)) and CI values (Table 4) represented that ferulic acid and Z-ligustilide synergistically inhibited cold-induced upregulation of TRPM8 expression.

TRPM8 and TRPA1 ion channel have been improved to play an important role in processing pain, but the role in cardiovascular system is still controversial. Role of TRPM8 in mediating vasoconstriction and vascular tone is full of paradox. On the one hand, cold-induced vasoconstriction in hind paw was reduced in TRPM8 KO mice or in WT mice pretreated with TRPM8 antagonist AMTB [5]; meanwhile, cold-induced contraction of rat gastric fundus can be attenuated by TRPM8 antagonist as well as ROCK antagonist Y-27632 [20]. On the other hand, Johnson et al. [12] proved that TRPM8 nonspecific agonist menthol caused moderate contraction in relaxed tail artery, but relaxation of vessels precontracted with KCL or phenylephrine. It is possible that TRPM8 channel in sympathetic terminals can cause release of neurotransmitters, resulting in vasoconstriction under relaxed condition. Besides, TRPM8 may be activated in sensory axon collateral nerves which can release vasodilator substances such as calcitonin gene-related peptide [21]. As far as TRPA1 was concerned, activation of TRPA1 could induced either vasoconstriction accomplished by norepinephrine and ROS [5] or vasodilation induced by vasodilator substance released from sensory nerve [17, 22].

Our results showed that both TRPM8 specific agonist WS-12 and TRPA1 specific agonist ASP 7663 could extent phosphorylation of MLC_20_, that is, stimulated HASMC contraction directly. However, some differences of contraction mechanisms existed. TRPA1-induced contraction was achieved by calcium-dependent and calcium-sensitization way, while TRPM8-induced contraction was achieved mainly through calcium-dependent way. As far as CICR was concerned, activation of TRPM8 could induce calcium released from ryanodine receptor while TRPA1 could activate both ryanodine receptor and IP_3_ receptor.

In the last part, we discussed cold-induced upregulation of TRPM8 and TRPA1. Both oxaliplatin-induced acute cold-hypersensitivity model and chronic construction injury (CCI) model of neuropathic pain in rat were accompanied with upregulation of TRPM8 and TRPA1 expression in dorsal root ganglia (DRG) [23–25]. Whether cold temperature can alter TRPM8 and TRPA1 expression in vascular system is still unknown. It is reported that phosphorylation of ERK and p38 in DRG appeared when the peripheral tissue was stimulated by cold temperature. Furthermore, immunohistochemistry proved that p-p38-IR neurons heavily colocalized with TRPA1, whereas the majority of p-ERK-IR neurons were positive for TRPM8 [26]. Cold-induced upregulation of p-ERK may contribute to upregulation of TRPM8. Our results indicated that cold stimulation could induce upregulation of TRPM8 but not TRPA1. In previous experiment, we discovered that amount of TRPA1 was much lesser than TPRM8 in human aortic smooth muscle cell. Expression of TRPA1 may not enough to be detected by qRT-PCR. Further investigation is needed.

*A. sinensis* has been used as a medical plant in numerous traditional Chinese medicine prescriptions for thousands of years. Previous studies showed that the effective constituents of A. sinensis extract could be classified into water-soluble parts and essential oil. One of the major constituents of water-soluble parts is ferulic acid, while Z-ligustilide has been reported as one of the major essential oil components [3, 4]. It is proved that Z-ligustilide can inhibit vasoconstriction induced by vasoconstrictors, such as norepinephrine and calcium chloride, on rat aortic segment. It may also protect ischemic brain injury in rats due to inhibiting the calcium influx through voltage-dependent calcium channel and receptor-mediated calcium [27]. Ferulic acid can induce concentration-dependence aorta vasodilation in chronic renal hypertensive rats [28]. It may also reduce carotid arterial pressure in anesthetized spontaneously hypertensive rats [29]. Synergistic inhibiting effect of these two components on calcium influx and combination inhibiting effect on RhoA-GTP induced by TRPM8 and TRPA1 agonist as well as synergistic inhibition on cold-induced upregulation of TRPM8 indicated that* A. sinensis* may attenuate, at least partially, vasoconstriction induced by cold-sensing channels TRPM8 and TRPA1.

## 4. Conclusion

Activation of TRPM8 and TRPA1 could induce contraction of human aortic smooth muscle cell via calcium influx and RhoA pathway. Cold temperature could upregulate TRPM8 expression. Synergistic effect on inhibiting calcium influx and cold-induced upregulation of TRPM8 by ferulic acid and Z-ligustilide was discovered.

## Figures and Tables

**Table 1:** Concentration of combination drug used in inhibiting [Ca^2+^]_i_ response to agonists.

Agonist	Ferulic acid (*μ*M)	Z-ligustilide (*μ*M)	Total concentration (*μ*M)
WS-12	2.1	2	4
	4.2	3.9	8.1
	16.8	15.6	33.1
	33.4	31.3	64.7

ASP 7663	2.3	1.7	4
	4.6	3.4	8
	18.3	13.7	32
	36.5	27.3	63.8

**Table 2:** Combination index (CI) at ED_70_, ED_75_, and ED_90_ values with drug combination on inhibiting [Ca^2+^]_i_ response to agonists. The CI values at Fa value of 0.70, 0.75, and 0.90 for isobolograms (Figure 3) were calculated with the Calcusyn version 2.1 software.

		ED_70_	ED_75_	ED_90_
WS-12	FA : lig (8.3 : 7.8)	0.16218	0.16971	0.22336
ASP 7663	FA : lig (9.1 : 6.8)	0.26528	0.2655	0.2848

**Table 3:** Concentration of single drugs and combination drug used in inhibiting cold-induced upregulation of TRPM8.

Single drug (*μ*M)		Combination drug (*μ*M)		Total concentration
Ferulic acid	Z-ligustilide	Ferulic acid	Z-ligustilide	
3.125	3.125	3.4	2.3	5.7
6.25	6.25	6.8	4.7	11.5
12.5	12.5	13.7	9.3	23
25	25	27.3	18.6	45.9

**Table 4:** Combination index (CI) at ED_70_, ED_75_, and ED_90_ values with drug combination on inhibiting cold-induced upregulation of TRPM8 expression. The CI values at Fa value of 0.70, 0.75, and 0.90 for isobolograms (Figure 5(c)) were calculated.

Drug (ratio)	CI values at		
	ED_70_	ED_75_	ED_90_
FA : lig (3.416 : 2.325)	0.14943	0.16879	0.28823
